# Role of Age-Related Shifts in Rumen Bacteria and Methanogens in Methane Production in Cattle

**DOI:** 10.3389/fmicb.2017.01563

**Published:** 2017-08-14

**Authors:** Chong Liu, Qinghui Meng, Yongxing Chen, Mengsi Xu, Min Shen, Rui Gao, Shangquan Gan

**Affiliations:** ^1^Institute of Environment and Sustainable Development in Agriculture, Chinese Academy of Agricultural Sciences Beijing, China; ^2^State Key Laboratory of Sheep Genetic Improvement and Healthy Production, Xinjiang Academy of Agricultural and Reclamation Science Shihezi, China; ^3^The Key Laboratory of Xinjiang Endemic and Ethnic Diseases and Department of Biochemistry, Shihezi University School of Medicine Shihezi, China

**Keywords:** enteric methane production, rumen microbiota, dairy cow, age-related microbiota, high-throughput sequencing

## Abstract

Rumen microbiota are essential for maintaining digestive and metabolic functions, producing methane as a byproduct. Dairy heifers produce large amounts of methane based on fermentation of digested organic matter, with adverse consequences for feed efficiency and the environment. It is therefore important to understand the influence of host age on the relationship between microbiota and methane production. This study explored the age effect on the relationship between microbial communities and enteric methane production in dairy cows and heifers using high-throughput sequencing. Methane production and volatile fatty acid concentrations were age-related. Heifers (9–10 months) had lower methane production but higher methane production per dry matter intake (DMI). The acetate:propionate ratio decreased significantly with increasing age. Age-related microbiota changes in the rumen were reflected by a significant shift in bacterial taxa, but relatively stable archaeal taxa. *Prevotella, Ruminococcus, Flavonifractor, Succinivibrio*, and *Methanobrevibacter* were affected by age. This study revealed different associations between predominant bacterial phylotypes and *Methanobrevibacter* with increasing age. *Prevotella* was strongly correlated with *Methanobrevibacter* in heifers; howerver, in older cows (96–120 months) this association was replaced by a correlation between *Succinivibrio* and *Methanobrevibacter*. This shift may account for the age-related difference in rumen fermentation and methane production per DMI.

## Introduction

Methane is one of the most abundant greenhouse gasses in the atmosphere, with a significant impact on global warming 28-times that of carbon dioxide (IPCC, 2014). According to estimation, anthropogenic methane emissions account for ∼20.7% total greenhouse gas emissions, and enteric methane emissions account for 30–40% of total anthropogenic methane emissions (IPCC, 2007). Ruminants are the largest source of methane emissions among livestock, releasing methane into the atmosphere through eructation. Methane production from ruminants is influenced by numerous factors, including diet, breed, genetics, and geographical range, which may have interactive effects on methane emission (Basarab et al., 2013; Carberry et al., 2014; Henderson et al., 2015; Zhao et al., 2015; Roehe et al., 2016).

These aforementioned factors have direct or indirect effects on rumen microbes that respond or adapt to environmental changes, potentially resulting in physiological changes in their host. In comparison to those factors, the age effect on microbial community and the physiological response from the animal host is rarely reported in ruminants. Two studies reported that human gut microbiota changed with age (Koenig et al., 2011; Yatsunenko et al., 2012). Age-related effects could thus result in hosts developing a specific model of microbiota and cooperating with them to produce effects and metabolism peculiar to the host, as reported for the family Christensenellaceae in the human gut (Goodrich et al., 2014). If we therefore assume that rumen microbiota respond or adapt to host age, this suggests that the taxa composition, and interactions among the microbes determining energy metabolism and methane production, may differ in host populations of different ages. Jami et al. (2013) explored the bovine rumen bacterial community from birth to adulthood (2-years-old), and concluded that they were influenced not only by diet, but also by the age of the host. However, the authors did not correlate changes in microbiota in different age groups with enteric methane production.

Methane production per kilogram of dry matter intake (DMI) is an important index for evaluating the potential methane production per head, and is also a marker reflecting the state of rumen fermentation. However, age-related changes in methane production per kilogram of DMI have rarely been investigated. Knight et al. (2008) found that methane production per kilogram of DMI was greater in 12–16-week-old calves compared with adult cows fed the same low-quality feed. Grandl et al. (2016) recently evaluated the trends in enteric methane production and feed efficiency across a wide age range of dairy cattle, and detected differential methane production in heifers and lactating cows. Their study revealed a curvilinear relationship between age and methane production, with methane production increasing with age in heifers (8–25 months) but decreasing in adult cows (4–10 years). We also previously measured enteric methane production in heifers and adult cows (Liu et al., 2016), using the sulfur hexafluoride (SF_6_) tracer technique, and showed an average of 35.1 ± 2.8 g/kg DMI for heifers (10 months) significantly higher than 27.2 ± 0.9 g/kg DMI for adults (3–4 years; *P* < 0.05). In contrast, another study reported no difference between < 1-year-old heifers and > 6-year-old cows fed ryegrass in terms of methane production per kilogram of DMI (Ramírez–Restrepo et al., 2015). However, Grandl et al. (2016) explained that discrepancies of methane production may be derived from improper or inaccurate divisions of age groups. Based on the above reports, we therefore intend to understand the age-related changes in methane production per kilogram DMI by studying changes in microbial communities. We hypothesized that age-related shifts or succession of microbes may be associated with the change of enteric methane production. We tested our hypothesis using high-throughput sequencing techniques to investigate the rumen bacterial and archaeal rumen communities in cows of different ages. This approach will help to identify the dominant bacterial and archaeal taxa, reveal their associations with enteric methane production, and understand the interactions among microbes in different age groups of cows.

Recent studies of rumen microbial communities in calves (<2 years old) reported the existence of age-related changes on the rumen microbiota (Li et al., 2011; Jami et al., 2013). However, the natural lifespan of a cow could be up to 25 years, and the average culling age for dairy cows worldwide is > 6 years, indicating that these existing data are not sufficiently comprehensive. In this study, we extend the investigated age range with the aim of determining the effect of age on enteric methane production, and relating this to the rumen microbial composition and associations in dairy cows of different ages.

## Materials and Methods

### Ethics Statement

This study was carried out in accordance with the Guidelines for the Care and Use of Laboratory Animals of the Scientific Research Department of Xinjiang Academy of Agricultural and Reclamation Sciences (protocol approval decision number: XJNKKXY-AEP-038). This study did not involve any endangered or protected animal species. Specific permissions with regard to the location or activities were not required because the study did not cause any harm to the experimental animals.

### Experimental Design and Feed Analysis

Twenty healthy female Holstein cattle of different ages were randomly selected and grouped by age as follows: S1 (heifers, *n* = 6, 9–10 months), S2 (young adults, *n* = 7, 45–65 months), and S3 (older adult, *n* = 7, 96–120 months). All animals were individually penned and fed the same total mixed ration diet based on corn silage, and were allowed to eat and drink freely. The animals used in this study were not genetically related or receiving antibiotic treatment. All adult cows in the S2 (5–7 month post-partum with average of 2–3 offspring) and S3 groups (6–7 month post-partum with average of 6–8 offspring) were lactating, with average milk yields of 23.6 and 24.9 kg/day per head, respectively. This dietary regime was designed by the farm administrators and had remained unchanged for at least 6 years. The ingredients and chemical composition of the diet are shown in Supplementary Table S1.

When the sampling began, the daily feed supply for each individual animal was calculated according to body weight and estimation during the adaptive phase, with an excess of 10% for each animal to meet their dietary requirements. The initial and residual feed samples for each animal were collected and weighted on 5 consecutive days to measure fresh feed intake. Both samples of fresh and residual feeds were dried to calculate DM%, then the difference value of total DM contents between supplied fresh diet and residual diet was DMI. The feed samples were dried in an oven at 105°C for 24 h, then ground through a 1-mm sieve before conventional feed analysis. Dry matter, neutral detergent fiber, acid detergent fiber, ether extract, ash, and total phosphorus were analyzed according to the China National Standards protocols (GB/T 6435–2014, GB/T 20806–2006, NY/T 1459–2007, GB/T 6433–94, GB/T 6438–2007 and GB/T 6437–2002). Gross energy was measured using a bomb calorimeter (Staufen, IKA, Germany). Crude protein was calculated as total nitrogen × 6.25. Total nitrogen and total carbon contents were analyzed using an element analyzer (Elementar, Heraeus, Germany).

### Enteric Methane Measurement

Enteric methane emissions were measured using the SF_6_ tracer technique, according to Johnson and Huyler (1994). Two weeks prior to experimental investigation, permeation tubes filled with 1.4–1.6 g of SF_6_ were placed into the rumen for equilibrium. The release rate from the permeation tube was determined by analyzing the tube weights over time (approximately 4.0 ± 0.2 mg/d per tube). After a 15-day adaptive phase, eructated gas was collected on 5 consecutive days. The sampling apparatus consisted of a U-shaped stainless steel vessel and a filter (50 μm) connected by a capillary tube to control gas-sampling time. The U-shaped canister was fixed around the neck, while the filter was placed near the cow’s mouth and nostrils. The filter was used to keep the capillary line from plugging. During use, the U-shaped vessel was first vacuumed, and the gas-sampling time was regulated by the valve and the length of capillary tube. After a 24-h collection period, the pressure inside the collection vessel was tested; if it was within the normal range, the vessel was flushed with pure N_2_ (99.9999%) to reach a final pressure of 0.15 MPa, and then transferred to gas-sampling bags prepared for gas chromatography analysis.

A gas chromatograph GC-14B (Shimadzu, Kyoto, Japan), fitted with an electron capture detector and a flame ionization detector, was used to measure SF_6_ and methane concentrations, respectively. The standard gasses of methane and SF_6_ were purchased from Standard Substances Center of National Institute of Metrology (Beijing, China) at concentrations of 1.02 × 10^2^ mg/L and 9.99 μg/L, respectively. The chromatographic conditions were as follows: oven temperature 80°C, column temperature 100°C, detector temperature 200°C, air-flow rate 50 mL/s, hydrogen-flow rate 60 mL/s, nitrogen-flow rate 270 mL/s and sample volume 1 mL. The methane output was calculated using the formula reported by Boadi et al. (2002) as follows: Q = R × C1/C2, where Q indicates methane yield per day, R indicates the SF_6_ release rate, and C1 and C2 indicate the methane and SF_6_ concentration in samples.

### Ruminal Fluid Analysis

The rumen contents were collected approximately 2–3 h after the morning feeding with a flexible plastic stomach tube, which was used to aspirate approximately 200 mL of ruminal contents, with the initial 100 mL discarded to avoid contamination by saliva and subsequent samples (∼100 mL) collected. The obtained sample was strained through four layers of cheesecloth and transferred to a sterile 10-mL centrifuge tube. The ruminal fluid samples were divided into duplicated aliquots and snap frozen in liquid nitrogen. One aliquot was used for microbial DNA extraction, and the other was used for measuring the concentrations of volatile fatty acids (VFAs) and ammonia-N. These samples were then transported to the laboratory and kept frozen at -80°C. Concentrations of VFAs were analyzed using a previously reported method (Hoskin et al., 1995). Ruminal fluid samples were centrifuged at 12,000 × *g* for 5 min at 4°C, and the supernatant from each sample was then transferred to a 2-mL tube. After adding 25% (w/v) metaphosphoric acid solution to the fluid at a volume ratio of 1:5, the mixture was vortexed for 10 s and centrifuged at 2000 × *g* for 20 min to remove the precipitate. The supernatant was then loaded on a GC-14B gas chromatograph (Shimadzu) to measure the main VFAs concentration. Ammonia-N concentration was determined by a colorimetric technique using a spectrophotometer (Hash DR 6000, Loveland, CO, United States) following the method of Chaney and Marbach (1962).

### Sequencing and Bioinformatics

Microbial DNA was extracted from the ruminal content samples using a QIAamp Fast DNA Stool Mini Kit (Qiagen, Dusseldorf, Germany). Sample was added into the lysing matrix that blended with mini Bead Beater (BioSpec, Bartlesville, OK, United States) at 5,000 oscillations per minute for 60 s break open cell walls and release nucleic acid material. After lysis, microbial DNA extraction was conducted according to the protocol of manufacturer’s instruction. Extracted DNA was then quantified and checked for purity at 260:280 nm on a ND1000 spectrometer (Nanodrop Technologies, Wilmington, DE, United States) to ensure a yield of > 20 ng/μL nucleic acid material. Amplification and sequencing of the hypervariable region of the 16S rRNA gene was performed using the validated, region-specific primers optimized for the Illumina MiSeq platform. The primers used for the bacterial 16S rRNA hypervariable region (V3–V4) were 343F (5′-TACGGRAGGCAGCAG-3′) and 798R (5′-AGGGTATCTAATCCT-3′) (Nossa et al., 2010), and the primers for archaeal 16S rRNA hypervariable region (V4–V5) were Arch519F (5′-CAGCMGCCGCGGTAA-3′) and Arch915R (5′-GTGCTCCCCCGCCAATTCCT-3′) (Teske and Sørensen, 2008). PCRs were performed in triplicate in a 20 μL mixture containing 4 μL of 5 × FastPfu Buffer, 2 μL of 2.5 mM dNTPs, 0.8 μL of each primer (5 μM), 0.4 μL of FastPfu Polymerase, and 10 ng of template DNA. The PCR products were gel purified using the AxyPrep DNA Gel Extraction Kit (Axygen Biosciences, Union City, CA, United States) according to the manufacturer’s instructions, and quantified using QuantiFluor^TM^-ST (Promega, Fitchburg, WI, United States). Purified amplicons were pooled in equimolar ratio and paired-end sequenced on an Illumina Miseq platform according to standard protocols. Raw sequence were 114,853 and 107,191 reads at sequence depth per sample for bacteria and Archaea, respectively, and quality trimmed using Mothur (version 1.35) with the following criteria, as reported previously (Kozich et al., 2013). We conformed with the required standard for reads processing: (i) sequences could not start with ambiguous bases; (ii) sequences could not contain homopolymers > 8 base pairs (bp); (iii) read lengths fell within 150–350 bp; and (iv) quality score of the base > 25 to be considered real. Chimeric sequences were identified and removed using chimera.uchime embedded in Mothur. The bacterial sequences were further classified using a Mothur-formatted version of the RDP training set with 1000 iterations to remove non-sense sequences, probably derived from residual food or contamination. The archaeal sequences were classified using RIM-DB according to previous report of Seedorf et al. (2014). High-quality aligned sequences were used to calculate uncorrected pairwise distances and were assigned to operational taxonomic units (OTUs, at 97% similarity). The OTU matrices were rarefied to 200 sequences per sample for bacteria and Archaea, and confirmed that sequencing of each sample was conducted to sufficient depth. Finally, obtained OTUs with singleton sequence across all samples were excluded from dataset, and converted to a table composed of samples (rows) and OTUs (columns) to allow further comparisons between age groups. Diversity indices were calculated using Mothur. Metastats were used to determine if any OTUs were differentially represented among samples from different age groups (White et al., 2009).

All bioinformatic analyses were performed using R packages. NMDS and similarity analysis were performed to show the separation of samples and test the significance of differences between the groups using the vegan package (Oksanen et al., 2017), based on the Bray–Curtis dissimilarity. The gplots package was used to plot Venn diagrams and heatmaps (Warnes et al., 2016), and the ggtern package was used to draw ternary diagrams representing the distribution of dominant microbes at OTU level among the three groups. The relationship between the microbial community composition, VFAs and methane output in different age groups was explored using ordination method. Because the axis length obtained by detrended correspondence analysis was < 3 (Lepx and Smilauer, 2003), a linear model of redundancy analysis (RDA) was used for further analysis of the correlations among samples, OTUs, and influencing variables. RDA was also performed with the vegan package to reveal the relationships among microbial taxa, samples, and variables and to test the significance of correlations between variables and microbial distribution. Network analysis was performed using the igraph package to present the age effect on microbial interactions (Csardi, 2015), and sPLS regression was performed to show OTUs related to response variables using the spls package (Chung et al., 2013). Phylogenetic trees were constructed using MEGA6 with 1000 replicates of bootstrap analysis, using the neighbor-joining method (Saitou and Nei, 1987).

### Real-Time PCR Analysis

Real-time PCR was carried out on an IQ5 detection system (Bio-Rad, San Diego, CA, United States), using SYBR green mixture reagent (Qiagen, Dusseldorf, Germany) in a volume containing 12.5 μL SYBR green mixture, 100 nM of each primer, and 1.0 μL template DNA. The primer pairs of uniMet1-F and uniMet1-R were used to amplify the partial methanogen 16S rRNA gene (Zhou et al., 2010), with the following amplification conditions: denaturation at 95°C for 10 min, followed by 40 cycles of denaturation at 95°C for 30 s, annealing at 56°C for 30 s, and extension at 72°C for 30 s. The primer pairs of Bac1 and Bac2 were used to amplify the partial bacterial 16S rRNA gene using the same conditions as above (Chin et al., 2010). The vector pGEM-T (Tiangen, Beijing, China) with inserted bacterial and archaeal fragments was used to amplify standard curves and determine copy numbers.

### Statistical Analysis

Analysis of Variance (ANOVA) with Bonferroni-corrected pairwise comparison was used to test for significant differences between age groups in terms of enteric methane emission, body weight, DMI, VFAs concentrations, ammonia-N concentration, microbial groups abundance. The abundance of dominant OTUs in age groups was compared using non-parametric Kruskal–Wallis test, because of lack of homogeneity of variances. Data were presented as bar plots and box plots using SPSS version 20 for Windows (IBM, New York, NY, United States).

### Nucleotide Sequence Accession Numbers

The sequences obtained from amplicon sequencing were deposited in the NCBI Sequence Read Archive under accession number SRP077855.

## Results

### Methane Production, Fermentation and Real-Time Polymerase Chain Reaction (PCR)

Feed intake, performance, and methane production in different age groups are shown in **Table 1**. Based on the same dietary regime, heifers (S1) produced the least methane, but showed the highest level of methane production calculated by DMI, which was significantly higher than in adult cows (S2 and S3; *p* < 0.05). There was no significant difference in methane production between the S2 and S3 groups (*p* = 0.105) (**Supplementary Figure S1**).

**Table 1:** Feed intake, performance, methane production, rumen fermentation parameters, and microbial population sizes in cattle in different age groups.

Item	Means ± SE			*P*-value
	S1 (*n* = 6)	S2 (*n* = 7)	S3 (*n* = 7)	
**Performance and methane production**
Body weight (kg)	321.33 ± 14.97^a^	663.14 ± 13.51^b^	686.43 ± 8.20^b^	0.001
DMI (kg/day)	6.37 ± 0.09^a^	16.15 ± 0.09^c^	15.25 ± 0.12^b^	0.010
CH_4_ (g/day/head)	191.93 ± 11.44^a^	379.02 ± 8.88^c^	313.73 ± 9.95^b^	0.001
CH_4_ (g/kg DMI)	30.13 ± 1.80^b^	23.47 ± 0.59^a^	20.57 ± 0.65^a^	0.001
CH_4_ (g/100 kg body weight)	60.34 ± 4.34^b^	57.33 ± 1.96^b^	45.66 ± 1.06^a^	0.002
**Rumen fermentation**				
Ammonia–N (mg/dL)	28.91 ± 1.95^b^	27.94 ± 1.82^b^	21.19 ± 2.24^a^	0.029
Acetate (mmol/L)	55.93 ± 7.56	65.69 ± 10.79	80.09 ± 7.11	0.183
Propionate (mmol/L)	14.31 ± 1.20^a^	19.00 ± 2.25^a^	29.47 ± 3.03^b^	0.001
Butyrate (mmol/L)	8.51 ± 1.74^a^	10.38 ± 2.10^ab^	14.08 ± 1.11^b^	0.094
Total VFA (mmol/L)	78.75 ± 10.40^a^	95.07 ± 16.16^ab^	123.62 ± 11.17^b^	0.054
A:P ratio	3.95 ± 0.52^b^	3.40 ± 0.22^ab^	2.79 ± 0.20^a^	0.071
**Microbial population size**				
Total methanogen (log copies/mL)	9.61 ± 0.14^a^	9.73 ± 0.08^ab^	9.92 ± 0.08^b^	0.124
Total bacteria (log copies/mL)	11.59 ± 0.11	11.78 ± 0.09	11.76 ± 0.13	0.446
Methanogen:bacteria proportion (%)	1.13 ± 0.19	1.07 ± 0.26	1.55 ± 0.27	0.340

*Within a row, different superscript letters indicate *p* < 0.05, and means without superscript letters indicate *p* > 0.05. A:P = acetate:propionate ratio. S1 indicates heifers (9–10 months); S2 indicates young adults (45–65 months); S3 indicates older adults (96–120 months).*

Basal fermentation parameters were further compared, including the concentrations of ammonia-N and the main VFAs in rumen fluid (**Table 1**). Ammonia-N concentration tended to decrease in older cows, with a significant difference between the S1 and S3 groups (*p* < 0.05), while acetate, propionate, and butyrate tended to increase in older cows, with significant differences in propionate and butyrate concentrations between the S1 and S3 groups (*p* < 0.05). The difference in acetate concentration between the groups was not significant (*p* = 0.183), though the difference between the S1 and S3 groups tended toward significance (*p* = 0.073). We further compared the acetate:propionate (A:P) ratio between the groups, and showed that this was significantly lower in S3 cows compared with S1 heifers (*p* < 0.05). We also quantified the total population sizes of methanogens and bacteria using real-time PCR (**Table 1**). The population of methanogens increased with age, with a significant difference between the S1 and S3 groups (*p* < 0.05), but there was no significant difference in the bacteria population size or methanogen:bacteria proportion (%) between the groups (*p* = 0.34).

### Rumen Bacterial Composition across Different Age Groups

After sequence processing, there were 108,864 sequences clustered into 3,504 OTUs for bacteria with an average of 5,743 ± 389 reads per sample, and 107,039 sequences clustered into 121 OTUs for Archaea with an average of 5,360 ± 1,132 reads per sample, based on 97% nucleotide sequence identity between reads. The numbers of shared and specific OTUs among groups are shown in a Venn diagram, and were more obvious for bacteria than Archaea (**Supplementary Figure S2**). The OTU numbers and taxon diversity (Shannon–Wiener diversity index) in bacteria differed significantly (*p* < 0.05) among the age groups, but there was no significant difference for Archaea (Supplementary Table S2).

Overall, 20 phyla were detected in the samples, among which Firmicutes, Bacteroidetes, and Proteobacteria were the most dominant phyla, accounting for > 92%–98.3% of total reads. The phyla Firmicutes and Proteobacteria did not vary significantly among the groups (*p* = 0.28). The incidence of the phylum Bacteroidetes was significantly lower in samples from the S3 group compared with the S1 and S2 groups (*p* < 0.05), attributable to the significant reduction in the genus *Prevotella*. The phyla Spirochaetes, Fibrobacteres, and Tenericutes were also compared between groups, and although they were not abundant, their concentrations in samples in at least one group were close to 1%. ANOVA detected significant differences in abundance among age groups for Fibrobacteres (*p* < 0.05) and Tenericutes (*p* < 0.01), while differences for the phylum Spirochaetes were almost significant (*p* = 0.098) (**Supplementary Figure S3**). Metastats analysis was used to compare the abundances of 482 bacterial genera among age groups, with a standard of > 0.5% abundance in at least one group. The genus composition of these phyla also changed; 14 genera affiliated to the phyla Firmicutes, Bacteroidetes, Spirochaetes, Fibrobacteres, and Tenericutes differed significantly among age groups (Supplementary Table S3). Though Proteobacteria accounted for 7.4%–11.8% of total reads, there were no significant differences in these taxa at the genus level among different age groups, indicating that the phylum Proteobacteria might occur irrespective of age.

We similarly analyzed the abundances of 17 archaeal genera in different age groups. A total of 16 genera affiliated to Euryarchaeota were detected, including 12 genera of methanogens. Among these methanogens, *Methanobrevibacter* and *Methanomassiliicoccus* accounted for > 99% of the total reads. The abundance of *Methanobrevibacter* and *Methanomassiliicoccus* ranged from 56.9 to 96.4% and 2.8 to 40.1% among samples, respectively. The relative abundances of methanogens were similar among different age groups (Supplementary Table S4).

### Microbial Diversity Analysis

**FIGURE 1:**
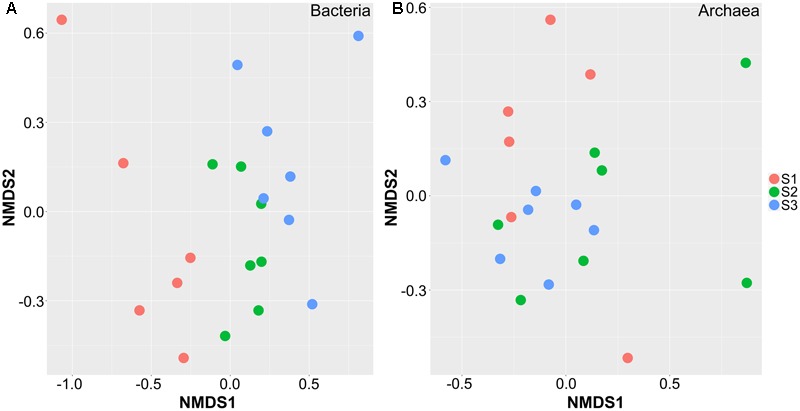
Non-metric multidimensional scaling plot showing the separation of samples from cattle in different age groups. These samples are separated based on the composition of OTUs in bacteria **(A)** and archaea **(B)**. S1 indicates heifers (9–10 months); S2 indicates young adults (45–65 months); S3 indicates older adults (96–120 months).

The bacterial and archaeal community OTUs were compared by non-metric multidimensional scaling (NMDS) using the Bray–Curtis dissimilarity metric, with a sequence identity ≥ 97% at the OTU level. The ordination plot showed apparent spatial separation among the samples based on bacterial communities (**Figure 1A**). The centers of the clouds representing the S2 and S3 groups were significantly separated from the S1 group using analysis of similarities (ANOISM; *p* < 0.05). Although S2 and S3 samples also showed a tendency to be separated, the centers of the clouds almost overlapped, indicating no significant difference between them (*p* = 0.31). The effect of age on sample separation could also be reflected by hierarchical clustering at the genus and OTU levels. The top 35 genera of bacteria and 100 OTUs of bacteria were selected to construct heatmaps, which also showed closer clustering of S2 and S3 samples at the OTU than at the genus level (**Supplementary Figure S4**). In comparison, there was no spatial separation between age groups according to the distribution of Archaea in the samples (*p* = 0.52), and all samples were allocated randomly on the plot (**Figure 1B**). The heatmaps also showed no obvious clustering according to age, similar to bacteria at the OTU level (**Supplementary Figure S5**). These results indicated that age had a greater effect on bacterial than on archaeal composition.

**FIGURE 2:**
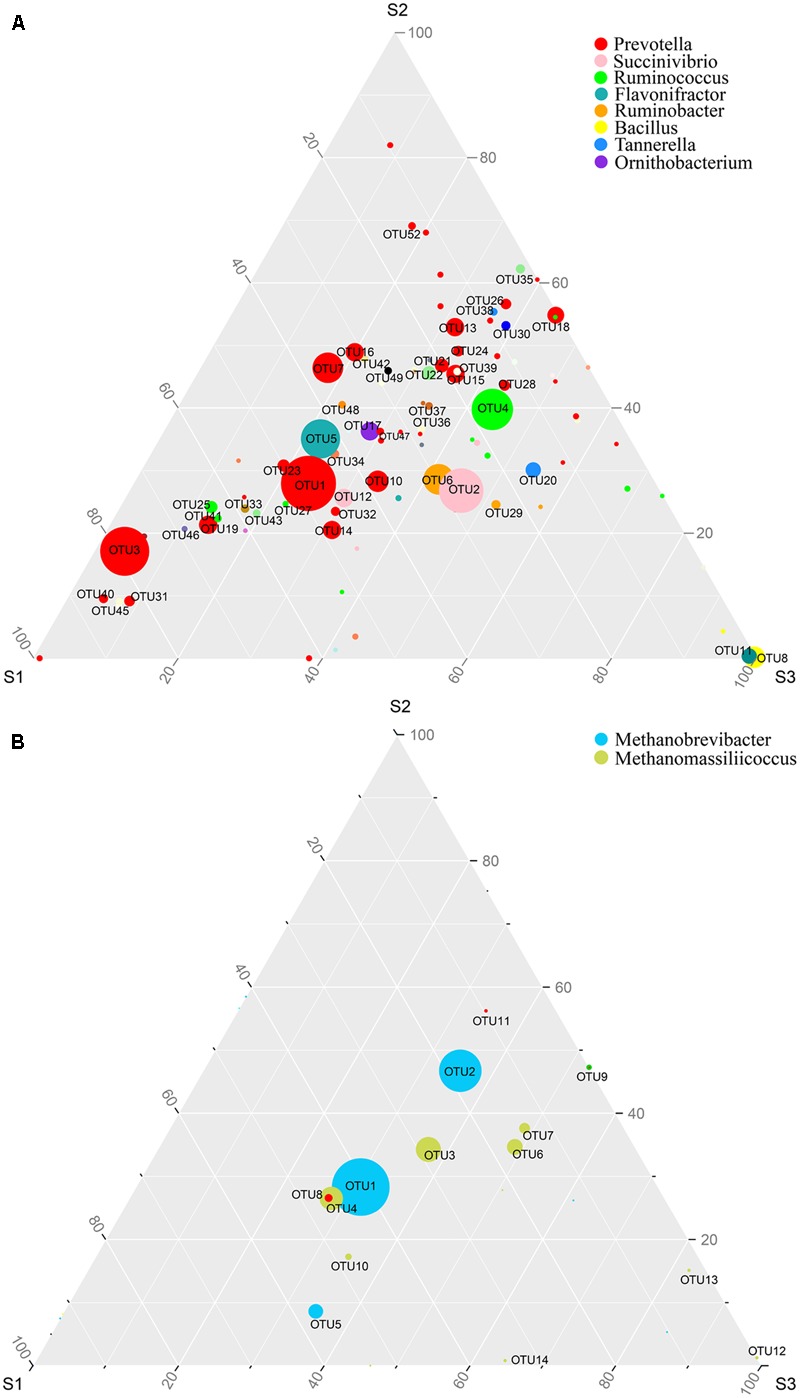
The distribution of microbial OTUs among different age groups. Ternary plot shows the comparative abundances of the top 100 bacterial taxa **(A)** and archaeal taxa **(B)** at OTU level shared among S1 (9–10 months), S2 (45–65 months), and S3 (96–120 months) age groups. The sum of the abundance for any one phylotype in these three groups is set as 100%. Symbol size reflects relative abundance of different genera.

We used ternary diagrams to track the distribution and relative abundance of microbial taxa at the OTU level in different age groups, and pairwise comparisons of taxa abundances were performed using Kruskal–Wallis tests. The abundance of some dominant OTUs increased or decreased with increasing age. *Prevotella* (OTU1, 3 and 7) changed greatly among age groups, and was significantly higher in S1 group (*p* < 0.05); *Succinivibrio* (OTU2) was significantly more abundant in S3 (*p* < 0.05); *Ruminococcus* (OTU4) was significantly more abundant in S2 and S3 (*p* < 0.05); and *Flavonifractor* (OTU5) was significantly more abundant in S1 (*p* < 0.05) (**Figure 2A** and **Table 2**). Regarding Archaea, there were only two dominant taxa at the phylotype level, affiliated to *Methanobrevibacter* (OTU1, 2 and 5) and *Methanomassiliicoccus* (OTU3, 4, 6 and 7). *Methanobrevibacter* accounted for > 78% of total Archaea, with relatively higher abundance of OTU1 in S1 (*p* < 0.05), and significantly higher abundance of OTU2 in S2 and S3 (*p* < 0.05). *Methanomassiliicoccus* accounted for 20.6% of total Archaea, with OTU3 averagely distributed, and relatively higher abundances of OTU4 in S1, and OTU6 and 7 in S2 and S3, with no significant difference among groups (**Figure 2B** and **Table 2**).

**Table 2:** Average abundance of dominant OTUs in cattle of different ages.

Domain	Genera	Phylotype	Average abundance (%)		
			S1 (*n* = 6)	S2 (*n* = 7)	S3 (*n* = 7)
Bacteria	*Prevotella*	OTU1	5.14 ± 1.26^b^	3.00 ± 0.57^ab^	2.45 ± 0.45^a^
		OTU3	6.29 ± 4.17^b^	1.76 ± 1.67^b^	0.41 ± 0.33^a^
		OTU7	2.10 ± 0.97^b^	2.64 ± 0.86^b^	0.96 ± 0.15^a^
		OTU15	0.63 ± 0.15^a^	1.44 ± 0.30^b^	1.03 ± 0.22^ab^
		OTU16	1.05 ± 0.14^ab^	1.66 ± 0.43^b^	0.71 ± 0.14^a^
		OTU18	0.01 ± 0.01^a^	1.60 ± 0.29^b^	1.28 ± 0.37^b^
		OTU19	2.33 ± 1.10^b^	0.71 ± 0.29^ab^	0.43 ± 0.06^a^
		OTU21	0.51 ± 0.17^a^	1.11 ± 0.14^b^	0.80 ± 0.07^ab^
	*Ruminococcus*	OTU4	1.45 ± 0.79^a^	3.03 ± 1.18^b^	3.17 ± 0.51^b^
	*Flavonifractor*	OTU5	3.43 ± 1.03^b^	2.64 ± 0.34^b^	1.72 ± 0.32^a^
	*Succinivibrio*	OTU2	2.45 ± 0.80	2.20 ± 0.49	4.21 ± 0.60
	*Ruminobacter*	OTU6	1.50 ± 0.81	1.66 ± 0.69	2.56 ± 1.91
	*Bacillus*	OTU8	0.00 ± 0.00	0.00 ± 0.00	3.05 ± 3.02
Archaea	*Methanobrevibacter*	OTU1	60.12 ± 9.59^b^	41.67 ± 5.96^a^	45.43 ± 5.82^ab^
		OTU2	14.27 ± 2.63^a^	37.12 ± 4.99^b^	28.04 ± 4.59^b^
		OTU5	4.70 ± 3.78	0.71 ± 0.18	2.85 ± 2.16
	*Methanomassiliicoccus*	OTU3	7.23 ± 3.40	8.67 ± 1.47	9.40 ± 1.85
		OTU4	10.03 ± 4.73	5.81 ± 1.20	6.08 ± 1.26
		OTU6	1.54 ± 0.78	3.24 ± 1.36	4.57 ± 1.28
		OTU7	0.51 ± 0.31	1.41 ± 0.50	1.83 ± 0.58
	*Methanosphaera*	OTU8	0.87 ± 0.37	0.50 ± 0.09	0.51 ± 0.12

*Comparison of OTUs between the groups was based on the test of homogeneity of variances, with non-parametric Kruskal–Wallis test to determine the significance. Within a row, means with different superscript letters indicate significant differences at *p* < 0.05, and means without superscript letter indicate *p* > 0.05. S1 indicates heifers (9–10 months); S2 indicates young adults (45–65 months); S3 indicates older adults (96–120 months).*

### Dominant Taxa Associating with VFAs and Methane Production

**FIGURE 3:**
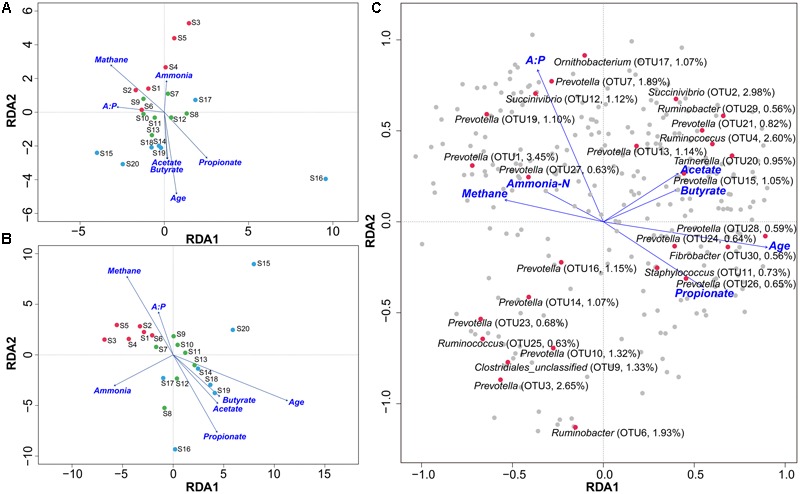
Effects of environmental variables on sample separation and microbial distribution using RDA. Bi–plot shows the relationships between samples and environmental factors, including ammonia–N, methane, A:P ratio, age, and VFAs at the genus **(A)** and phylotype **(B)** levels from cattle in the S1, S2, and S3 groups. Red dots indicate S1, green S2, and blue S3 groups. The relationships between predominant phylotypes and environmental factors are shown at phylotype level **(C)**. Red dots indicate predominant phylotypes. S1 indicates heifers (9–10 months); S2 indicates young adults (45–65 months); S3 indicates older adults (96–120 months).

**FIGURE 4:**
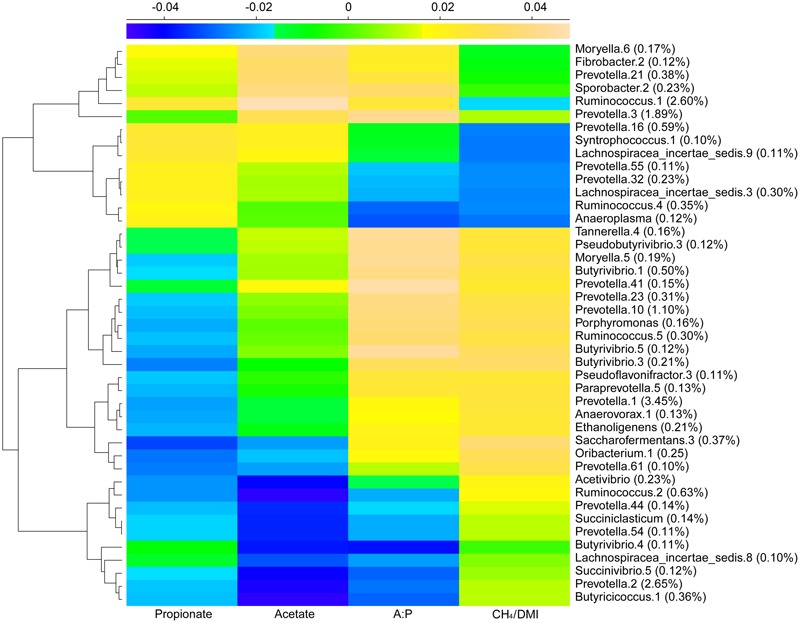
Dominant microbial OTUs related to VFAs and methane production using sPLS regression analysis. A heatmap based on coefficients represents the regression relationship between methane, VFAs and bacterial phylotypes with abundance > 0.1%.

Redundancy analysis method showed that, at either the genus or phylotype level, the constrained variables could explain about 39% of microbial distribution (**Figures 3A,B**). The bi-plot showed strong correlations in S1 samples between methane production and A:P ratio, and in S2 and S3 between primary VFAs and age, which were negatively correlated with methane production. We also depicted the distribution of the top 30 phylotypes with > 0.5% abundance in the samples (**Figure 3C**), which showed obvious separation along the gradients of variables. *Succinivibrio* (OTU2), *Ruminobacter* (OTU6 and 29), *Fibrobacter* (OTU30), *Ruminococcus* (OTU4), *Staphylococcus* (OTU11), and some *Prevotella* phylotypes with low abundance were scattered positively along the age gradient. In contrast, *Ornithobacterium* (OTU17), *Succinivibrio* (OTU12), and *Ruminococcus* (OTU25) with low abundance, and *Prevotella* phylotypes with high abundance (OTU1, 3, 7, 10, 14 and 16) were positively scattered along the gradient of methane production. The predominant genera of *Prevotella, Succinivibrio, Ruminococcus*, and *Ruminobacter* differed greatly at the phylotype level among the age groups. Statistical analysis of the variables determining bacterial distribution showed significant associations for methane output, age, and propionate concentration (Supplementary Table S5), with age having the highest determining coefficient (*R*^2^ = 0.69 and 0.84 at genus and phylotype levels, respectively), indicating that age was a key factor associated with the microbial distribution. These results were also supported by sparse partial least squares (sPLS) regression, in which 43 (>0.1% abundance) of the top 300 OTUs showed similar relationships with the response variables (e.g., methane production per DMI and VFAs) as presented by RDA (**Figure 4** and Supplementary Table S6).

### Co-occurrence and Network Analysis

**FIGURE 5:**
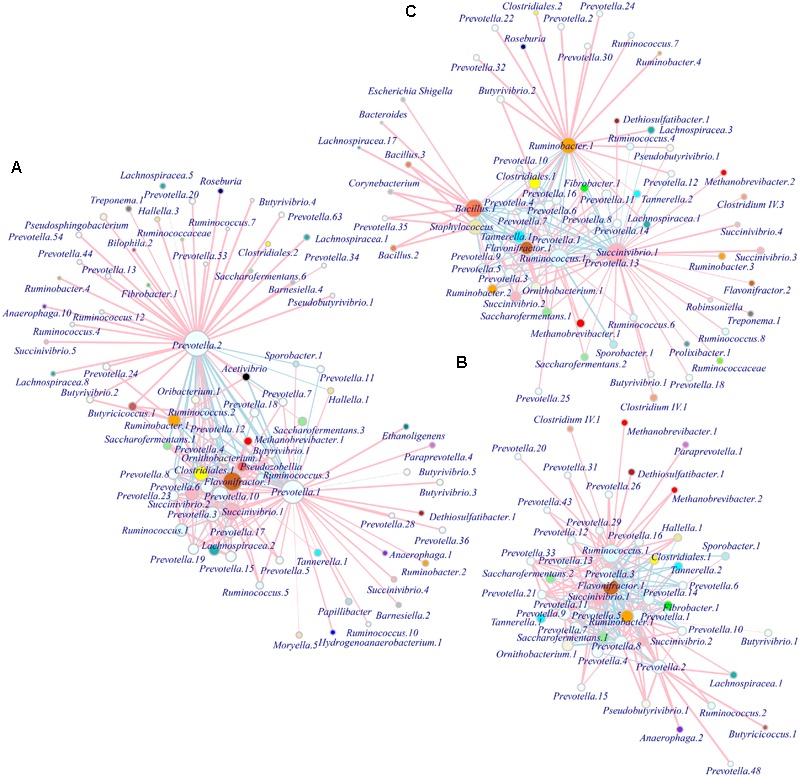
Patterns of microbial interactions in different age range. Networks are based on the adjacency matrix of microbial abundance and correlation analysis at the OTU level for groups S1 **(A)**, S2 **(B)**, and S3 **(C)**, respectively. The nodes represent microbial OTUs and the edges represent the correlation coefficients. The color and width of the lines indicate the direction (pink positive, light blue negative) and strength of the correlation between different microbial OTUs, respectively. The sizes of the nodes indicate the mean average abundance and the vertices representing OTUs within the same genus are colored the same. S1 indicates heifers (9–10 months); S2 indicates young adults (45–65 months); S3 indicates older adults (96–120 months).

We observed different modules of co-occurring OTUs in each group at the phylotype level, with the hubs connecting most other nodes being the phylotype *Prevotella.*1-2 (OTU1 and 3*)* in S1; *Prevotella.*1 (OTU1, 3 and 7), *Flavonifractor.*1 (OTU5), and *Ruminococcus.*1 (OTU4) in S2; and *Succinivibrio.*1 (OTU2), *Ruminococcus.*1, *Ruminobacter.*1 (OTU6), and *Bacillus.*1 (OTU8) in S3. These bacterial taxa predominantly occurred in different age groups (**Table 2**), forming their corresponding metabolic networks. Based on Ribosomal Database Project (RDP) classification and 16S rRNA sequences from the National Center for Biotechnology Information (NCBI), we used MEGA6 to construct trees of dominant OTUs using the neighbor-joining method (**Supplementary Figure S6**). Phylogenetic analysis showed the affiliations of the identified *Prevotella* phylotypes in this study, with dominant *Prevotella.*1 and 2 affiliated to *P. ruminicola*, and *Prevotella.*3 affiliated to *P. multiformis*. *Methanobrevibacter* phylotypes (*Methanobrevibacter.*1 and 2) were the only taxa related to bacteria in the network, and were identified with similarities to *M. thaueri*. *Ruminococcus* phylotypes (*Ruminococcus.*4 and 25) were found to be similar to some strains (e.g., *R. bromii* or *R. gauvreauii*). There was insufficient information in the database at the phylotype level to determine the affiliations for other genera, such as *Succinivibrio, Ruminobacter, Flavonifractor*, and *Bacillus*. In S1, *Prevotella.*1 (OTU1, *R* = 0.77), *Flavonifractor.*1 (OTU5, *R* = 0.78) and *Prevotella.*3 (OTU7, *R* = 0.59) were strongly correlated with *Methanobrevibacter.*1; while *Prevotella.*2 (*R* = -0.49) or *Prevotella.*10 (OTU19, *R* = 0.24) was negatively or weakly correlated with *Methanobrevibacter.*1 (**Figure 5A**). In S2, *Ruminococcus.*1 (OTU4) outcompeted other taxa and was positively correlated with *Methanobrevibacter.*1 (*R* = 0.41) and 2 (*R* = 0.69) and *Prevotella.*3 was positively correlated with *Methanobrevibacter.*2 (*R* = 0.68). In contrast to S1, the network in S2 seemed to be in a transition state with no overwhelming hubs (**Figure 5B**). In S3, the new hubs and network were shaped by dominant phylotypes of *Succinivibrio.*1 (OTU2) affiliated to Succinivibrionaceae, *Ruminobacter.*1, *Bacillus.*1, and *Ruminococcus.*1.*Succinivibrio.*1 was positively correlated with *Methanobrevibacter.*1 (*R* = 0.31) and 2 (*R* = 0.37), respectively; *Prevotella.*1 (*R* = 0.56) and *Ruminococcus.*1 (*R* = 0.29) were positively correlated with *Methanobrevibacter.*1 (**Figure 5C**).

## Discussion

The effect of age on methane production was previously investigated in ruminants, including cattle and deer (Molano et al., 2006; Swainson et al., 2007; Jiao et al., 2014), and was considered likely to be related to animal characteristics, such as body weight, DMI, and retention time (Okine et al., 1989; Moss et al., 2000). At the same time, age and physiological status were also reported to have effects on shifts in the rumen microbiome (Kumar et al., 2015; Pitta et al., 2016; Wang et al., 2016; Dill-McFarland et al., 2017). However, a comprehensive study of age effects on methane production and related changes in microbial communities and interactions was lacking.

The results of the current study showed higher methane production per DMI in heifers aged 9–10 months compared with adults aged 45–65 and 96–120 months. Therefore, differences in methane production based on DMI across a wide age range in populations of dairy cows are mainly influenced by developmental physiological changes, especially related to rumen fermentation, which responds to or is adapted by shifts in microbial communities. It is therefore important to determine the effects of age on rumen fermentation and the microbial community to improve our understanding of age-related changes in methane production. The concentration and profile of VFAs is related to methane production, as confirmed in previous studies (Pickering et al., 2015). For example, propionate serves as an alternative reducing equivalent sink relative to methane (Moss et al., 2000; Denman et al., 2015), and a low A:P ratio related to low methane production (Morgavi et al., 2008, 2013). In the present study, VFAs concentrations increased and A:P concentration ratio decreased with increasing age, with a significant difference between heifers and adult cows. This suggests that the metabolic pathways related to VFAs production might change, indirectly suggesting divergent microbiota in different age groups. Given that cows of all ages were fed the same diet, we suggest that these differences in microbial composition were caused by age-related physiological changes, thus highlighting the need to reveal the dominant methane-producing taxa and their interactions at different ages.

Inhibiting methanogen populations has been a major focus of methane mitigation in ruminants; however, the roles of specific bacteria and other microbes have been less well-studied. Recent decreases in the cost of sequencing, and the development of complex statistical models, have facilitated the creation of a comprehensive perspective by revealing the associations of various microbes and their interactions in the rumen. Some recent studies have reported on the co-occurrence of bacteria, Archaea, fungi or ciliates that has associations with the physiological status in hosts (Fouts et al., 2012; Lee et al., 2012; Kittelmann et al., 2013; Li et al., 2015). These studies shed new light on the roles of microbial cooperation within the same ecological niche. However, these studies largely concentrated on diet or host effects on taxa identification, rather than on age effects, and discussions on microbial taxa and their interactions were not related to methane production. The present study thus provides the first report to evaluate the effect of age on methane production and microbiota. We confirmed a shift in microbial taxa in relation to age, and categorization by methane production showed obvious separation using ordination analysis. Metastats analysis further revealed significant differences in the genera *Prevotella, Ruminococcus, Saccharofermentans, Butyrivibrio, Acetivibrio, Tannerella, Treponema*, and *Fibrobacter* and their affiliating phylotypes among host age groups (*P* < 0.05). In contrast, fewer Archaea were affected by host age, because of the smaller number of OTUs identified and the overwhelming dominance of *Methanobrevibacter* (OTU1 and 2) and *Methanomassiliicoccus* (OTU3 and 4), which together accounted for approximately 96% of all methanogens. The similar case was also reported in goat rumen that age has a more significant effect on bacteria than on Archaea (Wang et al., 2016). Although the abundance of multiple phylotypes showed differences among the age groups, only *Methanobrevibacter.*1 and *Methanobrevibacter.*2 showed significant differences in abundance. The switch in abundance of phylotypes also supported a relatively weak effect of age on the methanogen community.

Based on the age-related shift in microbial taxa, we inferred that the metabolic networks of microbes in the rumen might also differ with age. This was indirectly supported by increasing VFAs concentration and a decreasing A:P concentration ratio revealed by analysis of rumen fermentation. Exploring the composition and interaction of bacteria and methanogens at the phylotype level could thus help to further our understanding of the mechanisms of age-differential methane production. We observed different modules of co-occurring OTUs in each age group, and changing interactions among phylotypes with increasing age. The obvious aspect in networks showed gradual replacement of *Prevotella* as a hub with increasing age (e.g., *Prevotella.*2 was absolutely replaced), and the formation of new hubs of *Succinivibrio.*1, *Ruminobacter.*1, *Ruminococcus.*1 and *Bacillus.*1 in S3.*Prevotella* and *Succinivibrio* are both able to produce propionate as a major fermentation product, previously reported in other rumen studies (O’Herrin and Kenealy, 1993; Pinares-Patino et al., 2003; Caporaso et al., 2010; Purushe et al., 2010; Henderson et al., 2013). The production of propionate competes with methanogens for metabolic hydrogen consumption (Denman et al., 2015). Under these conditions, the more propionate produced, the less methane would be emitted with increasing age. However, it seems contradictory that some phylotypes of *Prevotella* (OTU1 and 3) were abundant in S1 and S2 hosts with lower propionate concentrations. Ordination analysis in this study showed that these two OTUs were distributed along the reverse direction of the propionate gradient, suggesting that they were less-related to propionate concentration; sPLS regression also revealed positive coefficients for methane production and negative ones for VFAs, but a reverse trend for *Succinivibrio.*1. The possible reasons for this apparent discrepancy might be attributed to differential propionate-producing abilities at the phylotype level or changing metabolic pathways, causing metabolic divergence of *Prevotella* at the phylotype level along the same environmental gradient. A similar example of rumen bacteria in relation to methane production in sheep was recently reported, and *Prevotella bryantii* was regarded as a marker of low-methane ruminotypes, while other phylotypes of *Prevotella* were related to high-methane ruminotypes (Kittelmann et al., 2014). We therefore speculate that *Succinivibrio.*1 might be important in lowering the rumen A:P ratio in older adult cows (S3 group), and the age-related shift in bacterial phylotypes from *Prevotella.*1 to *Succinivibrio.*1 may largely explain the differences in methane-production efficiency across a wide range of ages.

Other bacterial taxa could potentially influence rumen VFAs or methane production (e.g., *Flavonifractor, Ruminococcus*, and *Ruminobacter*). The abundance of *Flavonifractor.*1 (OTU5) decreased gradually with increasing age, with a significant reduction in abundance in S3 (*P* < 0.05, ANOVA). *Flavonifractor* has a weak capacity for sugar fermentation and utilization, with acetic and butyric acids as the major metabolic end products (Carlier et al., 2010). In our current study, *Flavonifractor.*1 was classified as the only key phylotype, and was only positively correlated with *Methanobrevibacter* in the S1 group, indicating that this correlation was age-related. *Ruminococcus* is one of the primary degraders of plant fiber in the rumen (Flint, 1997). In our study, although its abundance increased with age, its role as a hub was not obvious, making an association between the shift in *Ruminococcus* and age with methane production uncertain. *Ruminobacter* was also affiliated to Succinivibrionaceae and produced succinate as an end product (Nossa et al., 2010), though the average abundance was higher in the S3 group, and it was subject to strong individual variation. Succinivibrionaceae growth in the rumen was previously suggested to reduce methane emission, given that some members of this family could utilize hydrogen and thus act as competitors of methanogens (McCabe et al., 2015). Recent reports show that the bacterial community composition was different between primiparous and multiparous cows, indicating that the microbiome continues to evolve and mature with age (Kumar et al., 2015; Pitta et al., 2016). These reports, together with our study, imply that the change of microbiome with age might have a direct influence on animal physiological parameters and productivity. Further studies are needed to determine the metabolic mechanism responsible for these correlations by performing metagenomic and metatranscriptomic studies. Additionally, real-time PCR analysis showed no obvious changes in methanogen population sizes and values relative to the total bacterial population size among age groups, suggesting that the distinct interactions between *Methanobrevibacter* phylotypes and their corresponding bacteria might play important roles in methane production.

Overall, comparisons of rumen bacterial and archaeal communities and methane production in dairy cows of different ages suggested that the succession and interactions of microbial populations influence enteric methane production based on per kilogram of DMI. The present findings provide the basis for further investigations of the effects of microbe-host interactions on digestive and metabolic activities as the physiological base of age-related effects.

## Conclusion

The present study compared methane production among cows of different ages and observed significant age-related shifts and distributions of bacteria. Different patterns of interactions *between predominant bacteria and Methanobrevibacter* associated with the changes in methane production based on DMI in relation to age. The shift from *Prevotella* to *Succinivibrio* might play an important role in this process.

## Author Contributions

CL and SG conceived and designed the experiments. CL conducted the sequencing data analysis. QM, YC, MX, and MS contributed to the sampling and conducted the experiment. CL and RG wrote the first draft and SG critically reviewed the manuscript.

## Conflict of Interest Statement

The authors declare that the research was conducted in the absence of any commercial or financial relationships that could be construed as a potential conflict of interest.

## Supplementary Material

FIGURE S1 Methane production in relation to age group in cattle. Methane production (g) based on per DM intake **(A)** or **(B)** per head. *Y*-axis indicates methane production, and *x*-axis indicates month age of cattle. S1 indicates heifers (9–10 months); S2 indicates young adults (45–65 months); S3 indicates older adults (96–120 months).

FIGURE S2 Shared and unique OTUs of rumen microbes. Venn diagrams show the numbers of shared and unique OTUs (cutoff = 0.03) of bacteria and Archaea among S1 (9–10 months), S2 (45–65 months), and S3 (96–120 months) age groups of cattle.

FIGURE S3 Rumen compositions at phylum level in cattle in different age groups. Bar plot showing average bacterial phylum distribution (left panel). Box plot showing relative abundances of Bacteroidetes, Firmicutes, and Proteobacteria (upper right panel). Box plot showing relative abundances of Spirochaetes, Fibrobacteres, and Tenericutes (lower right panel). Boxes represent the interquartile range between the first and third quartiles and the vertical line inside the box indicates the median. Different letters to the right of the whiskers indicate significant difference at *p* < 0.05 using ANOVA analysis with Bonferroni–corrected pairwise comparison. S1 indicates heifers (9–10 months); S2 indicates young adults (45–65 months); S3 indicates older adults (96–120 months).

FIGURE S4 Heatmap diagram showing the top 35 genera and 100 OTUs of bacteria clustered among 20 samples grouped by host age. The data matrix is constructed based on Euclidean distance. Samples are marked numbers from 1 to 6 in S1 (9–10 months), 7 to 13 in S2 (45–65 months) and 14 to 20 in S3 (96–120 months). The *x*-axis shows values of abundance that is presented with different colors. Number behind name of phylotype indicates the OTU order, and name without number indicates the single OTU in the corresponding genus.

FIGURE S5 Heatmap diagram showing all genera and top 30 OTUs of Archaea clustered among 20 samples grouped by host age. The data matrix is constructed based on Euclidean distance. Samples are marked numbers from 1 to 6 in S1 (9–10 months), 7 to 13 in S2 (45–65 months) and 14 to 20 in S3 (96–120 months). The *x*-axis shows values of abundance that is presented with different colors. Number behind name of phylotype indicates the OTU order, and name without number indicates the single OTU in the corresponding genus.

FIGURE S6 Phylogenic analysis of partial 16S rRNA genes of *Prevotella, Methanobrevibacter*, and *Ruminococcus* at phylotype level. The trees are constructed using the neighbor-joining method with bootstrap values from 1000 replications. The scale bar equals an average of five nucleotide substitutions per 100 positions.
