# Supplementary figures and images for: Role of Age-Related Shifts in Rumen Bacteria and Methanogens in Methane Production in Cattle
Source: Front Microbiol. 2017 Aug 14;8:1563. doi: 10.3389/fmicb.2017.01563 (PMC5557790; doi:10.3389/fmicb.2017.01563)

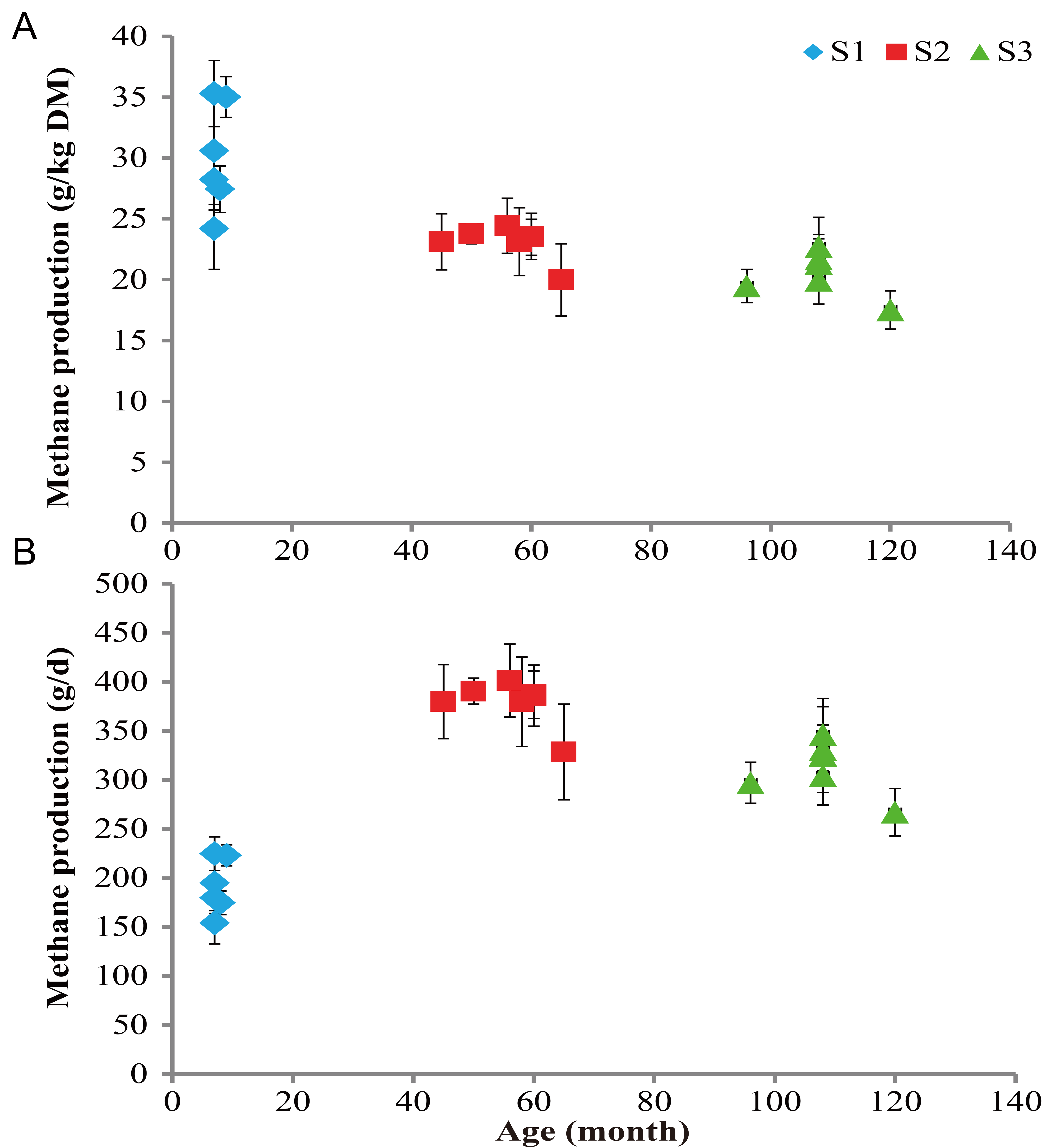

Supplement: FIGURE S1 — Methane production in relation to age group in cattle. Methane production (g) based on per DM intake (A) or (B) per head. Y-axis indicates methane production, and x-axis indicates month age of cattle. S1 indicates heifers (9–10 months); S2 indicates young adults (45–65 months); S3 indicates older adults (96–120 months). [file Image_1.TIF]

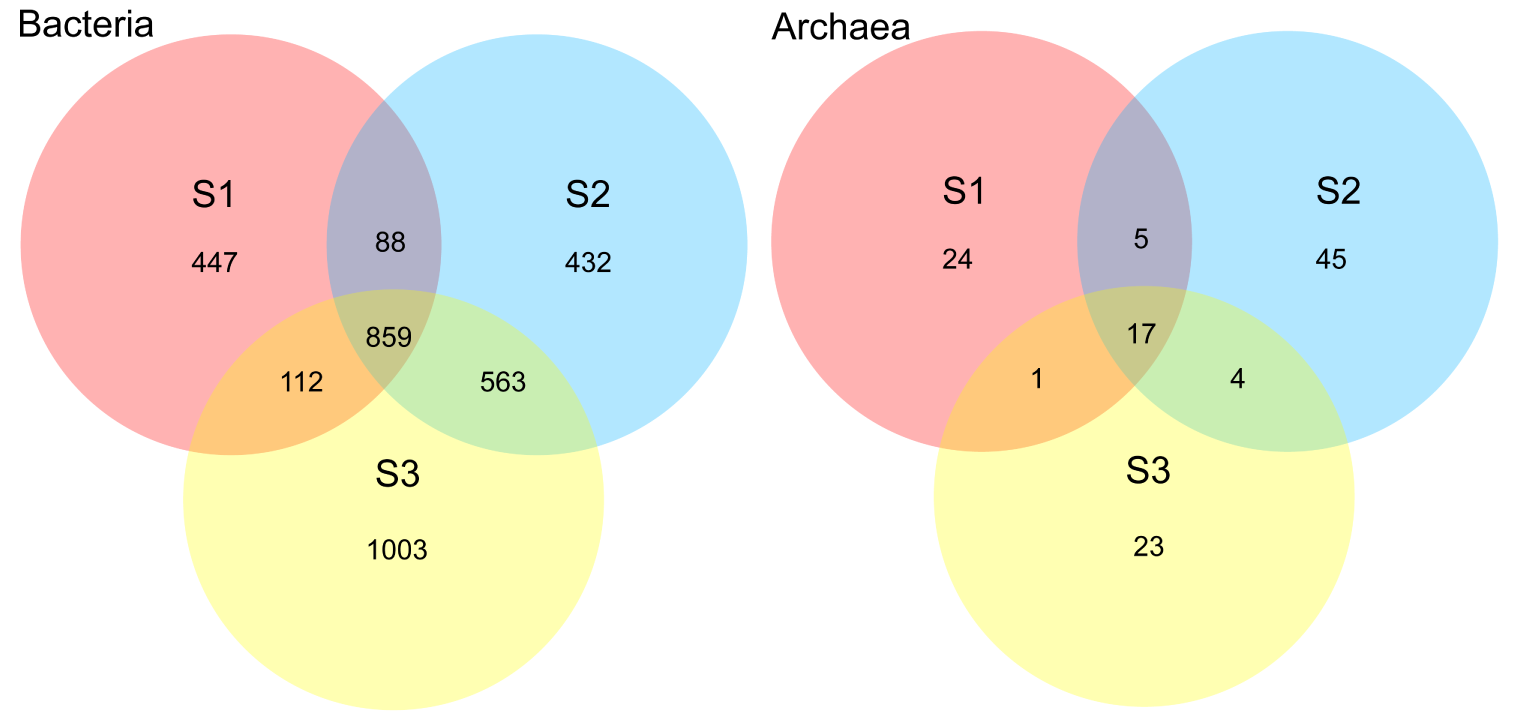

Supplement: FIGURE S2 — Shared and unique OTUs of rumen microbes. Venn diagrams show the numbers of shared and unique OTUs (cutoff = 0.03) of bacteria and Archaea among S1 (9–10 months), S2 (45–65 months), and S3 (96–120 months) age groups of cattle. [file Image_2.TIF]

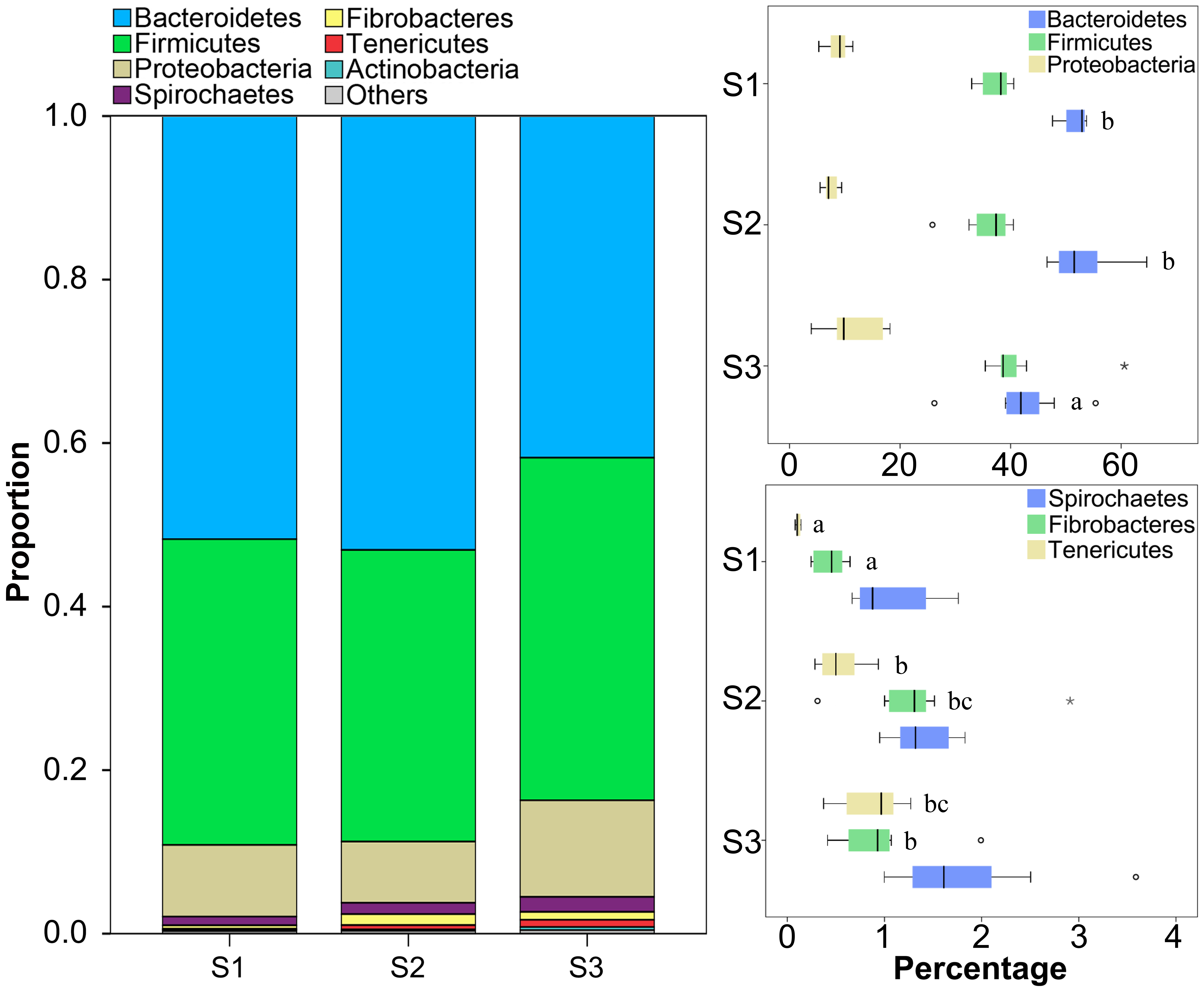

Supplement: FIGURE S3 — Rumen compositions at phylum level in cattle in different age groups. Bar plot showing average bacterial phylum distribution (left panel). Box plot showing relative abundances of Bacteroidetes, Firmicutes, and Proteobacteria (upper right panel). Box plot showing relative abundances of Spirochaetes, Fibrobacteres, and Tenericutes (lower right panel). Boxes represent the interquartile range between the first and third quartiles and the vertical line inside the box indicates the median. Different letters to the right of the whiskers indicate significant difference at p < 0.05 using ANOVA analysis with Bonferroni–corrected pairwise comparison. S1 indicates heifers (9–10 months); S2 indicates young adults (45–65 months); S3 indicates older adults (96–120 months). [file Image_3.TIF]

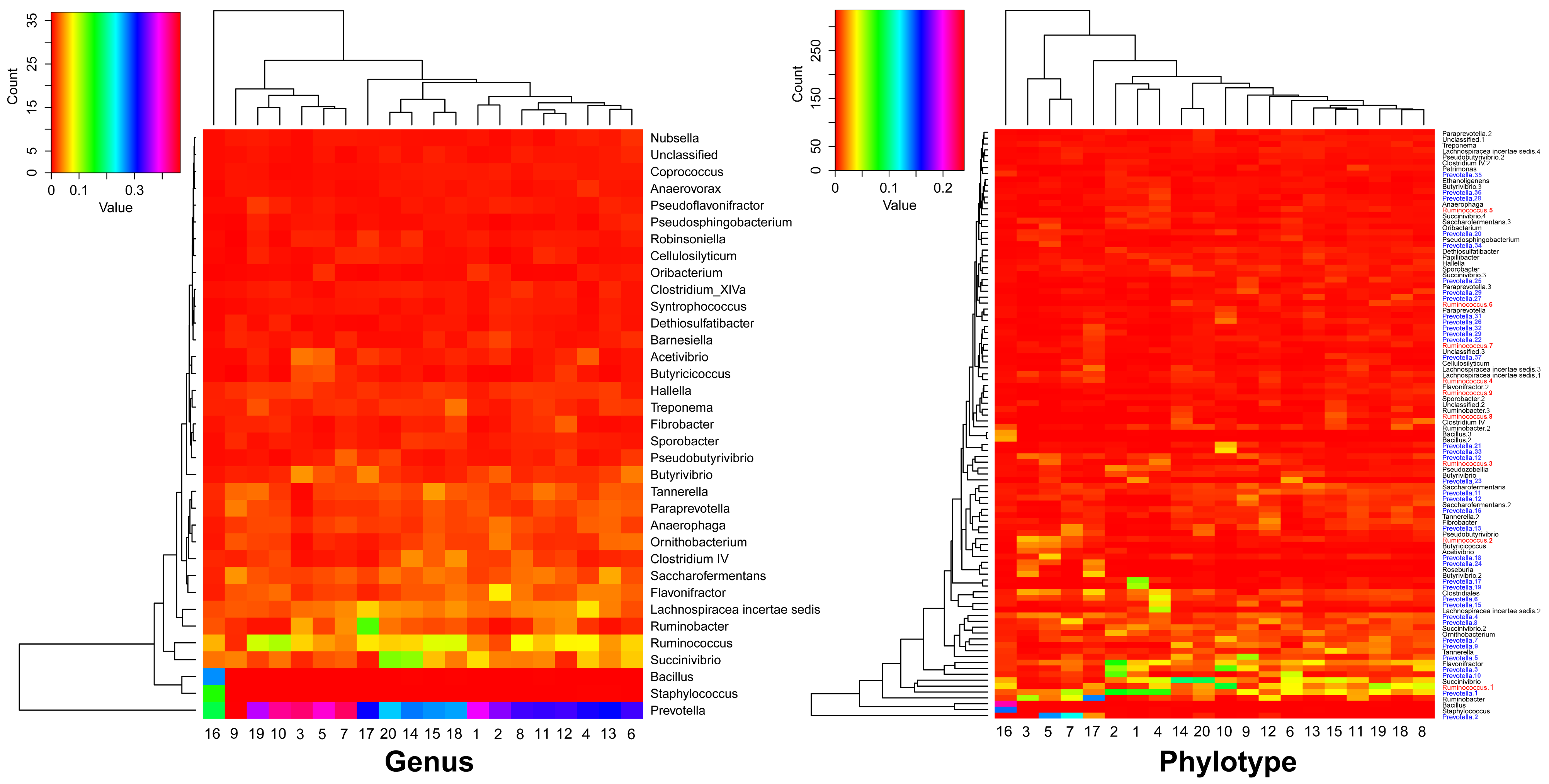

Supplement: FIGURE S4 — Heatmap diagram showing the top 35 genera and 100 OTUs of bacteria clustered among 20 samples grouped by host age. The data matrix is constructed based on Euclidean distance. Samples are marked numbers from 1 to 6 in S1 (9–10 months), 7 to 13 in S2 (45–65 months) and 14 to 20 in S3 (96–120 months). The x-axis shows values of abundance that is presented with different colors. Number behind name of phylotype indicates the OTU order, and name without number indicates the single OTU in the corresponding genus. [file Image_4.TIF]

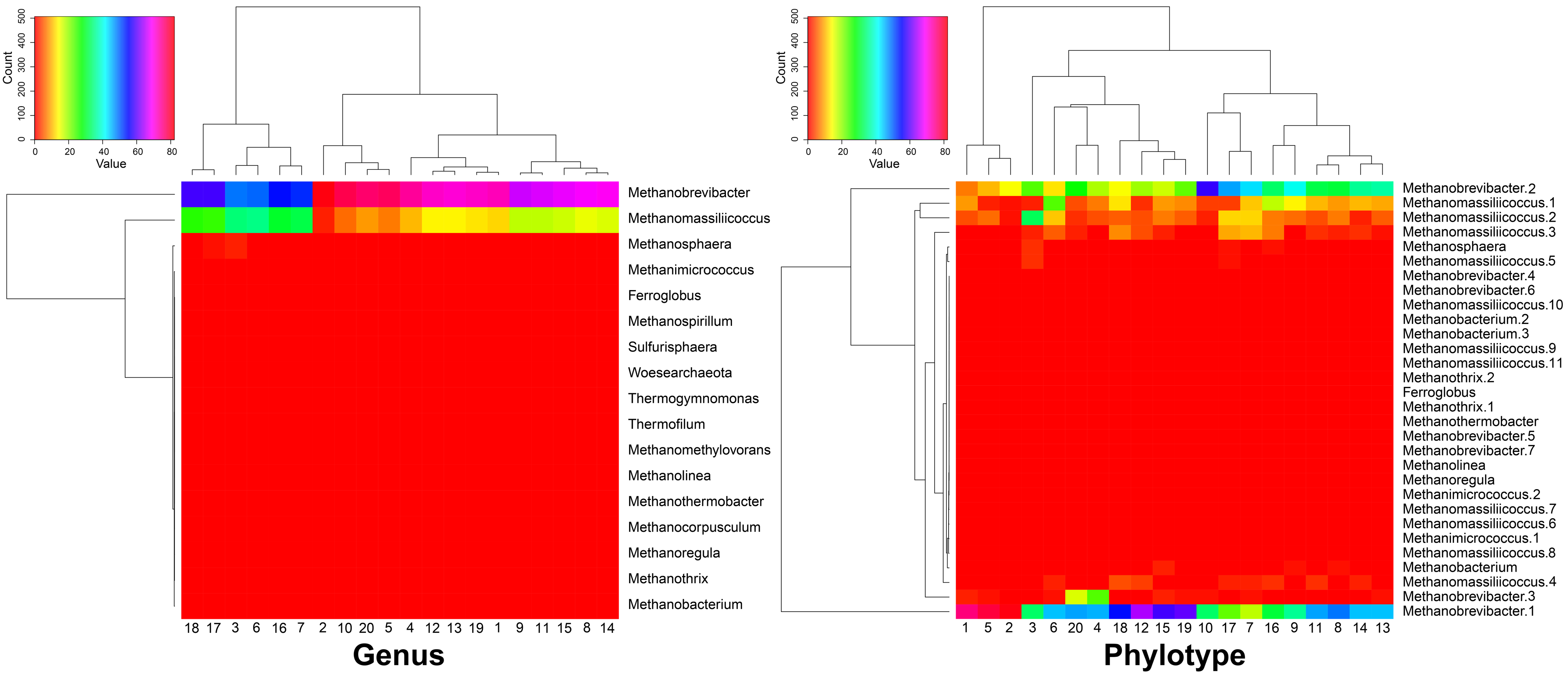

Supplement: FIGURE S5 — Heatmap diagram showing all genera and top 30 OTUs of Archaea clustered among 20 samples grouped by host age. The data matrix is constructed based on Euclidean distance. Samples are marked numbers from 1 to 6 in S1 (9–10 months), 7 to 13 in S2 (45–65 months) and 14 to 20 in S3 (96–120 months). The x-axis shows values of abundance that is presented with different colors. Number behind name of phylotype indicates the OTU order, and name without number indicates the single OTU in the corresponding genus. [file Image_5.TIF]

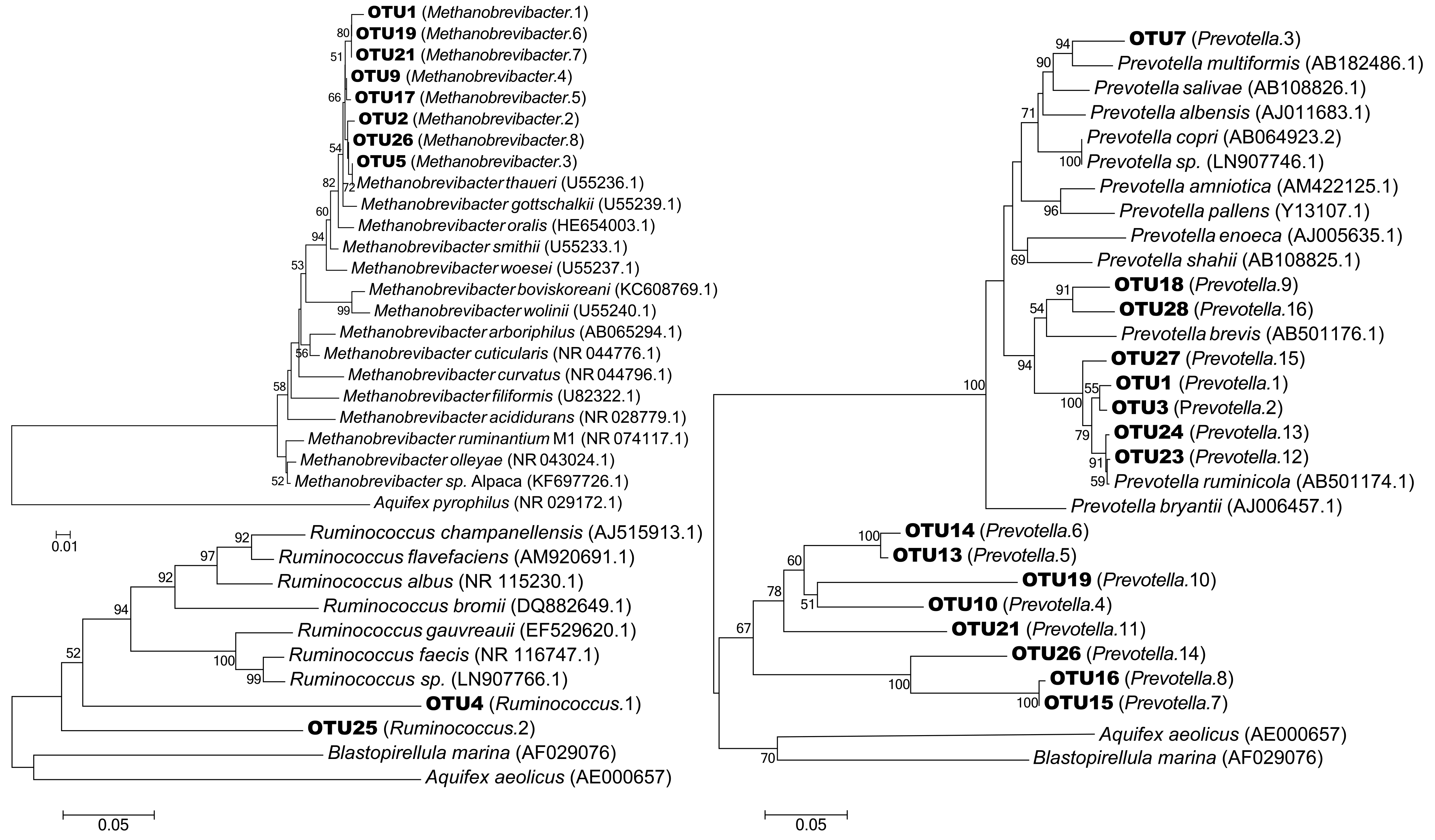

Supplement: FIGURE S6 — Phylogenic analysis of partial 16S rRNA genes of Prevotella, Methanobrevibacter, and Ruminococcus at phylotype level. The trees are constructed using the neighbor-joining method with bootstrap values from 1000 replications. The scale bar equals an average of five nucleotide substitutions per 100 positions. [file Image_6.TIF]
