# Supplementary material for: Role of Age-Related Shifts in Rumen Bacteria and Methanogens in Methane Production in Cattle
Source: Front Microbiol. 2017 Aug 14;8:1563. doi: 10.3389/fmicb.2017.01563 (PMC5557790; doi:10.3389/fmicb.2017.01563)
Supplement: Supplementary file 7 [file Table_1.DOC]

**Table S1. Ingredients and chemical composition of diet used in the experiment.** The composition of diet is based on DM. S1 indicates heifers (9–10 months); S2 indicates young adults (45–65 months); S3 indicates older adults (96–120 months).

| Item | Diet | | |
| --- | --- | --- | --- |
|  | S1 (n = 6) | S2 (n = 7) | S3 (n = 7) |
| Ingredient, % of fresh matter | | | |
| Corn silage | 67.3 | 67.3 | 67.3 |
| Alfalfa | 8.0 | 8.0 | 8.0 |
| Concentrate | 22.6 | 22.6 | 22.6 |
| Premix | 1.8 | 1.8 | 1.8 |
| Baking soda | 0.3 | 0.3 | 0.3 |
| Chemical composition, % of dry material | | | |
| Gross energy, MJ·kg^-1^ | 21.4 | 21. 3 | 21.5 |
| Crude protein | 14.3 | 15.2 | 14.8 |
| Ether extract | 2.7 | 2.8 | 2.8 |
| Neutral detergent fiber | 52.9 | 51.0 | 51.4 |
| Acid detergent fiber | 24.4 | 24.1 | 24.4 |
| Dry matter | 35.3 | 34.8 | 35.2 |
| Ash | 7.3 | 7.0 | 6.6 |
| Total phosphorus | 1.4 | 1.5 | 1.5 |
| Total carbon | 44.5 | 44.3 | 44.3 |
