# Supplementary material for: Role of Age-Related Shifts in Rumen Bacteria and Methanogens in Methane Production in Cattle
Source: Front Microbiol. 2017 Aug 14;8:1563. doi: 10.3389/fmicb.2017.01563 (PMC5557790; doi:10.3389/fmicb.2017.01563)
Supplement: Supplementary file 8 [file Table_2.DOC]

**Table S2. Bacterial and archaeal diversities between samples from different age groups of cattle.** S1 indicates heifers (9–10 months); S2 indicates young adults (45–65 months); S3 indicates older adults (96–120 months).

| Group | Bacteria | | | | Archaea | | | |
| --- | --- | --- | --- | --- | --- | --- | --- | --- |
|  | OTU No. | Shannon | Chao | Simpson | OTU No. | Shannon | Chao | Simpson |
| S1 (n = 6) | 598 ± 21^a^ | 4.9 ± 0.1^a^ | 961 ± 47^a^ | 0.03 ± 0.01 | 17 ± 2 | 1.1 ± 0.2 | 44 ± 19 | 0.47 ± 0.09 |
| S2 (n = 7) | 764 ± 46^b^ | 5.5 ± 0.1^b^ | 1228 ± 64^b^ | 0.01 ± 0.00 | 21 ± 4 | 1.3 ± 0.1 | 39 ± 11 | 0.36 ± 0.03 |
| S3 (n = 7) | 944 ± 59^c^ | 5.4 ± 0.2^b^ | 1533 ± 87^b^ | 0.02 ± 0.01 | 15 ± 1 | 1.4 ± 0.1 | 26 ± 6 | 0.34 ± 0.02 |
