# Supplementary material for: Role of Age-Related Shifts in Rumen Bacteria and Methanogens in Methane Production in Cattle
Source: Front Microbiol. 2017 Aug 14;8:1563. doi: 10.3389/fmicb.2017.01563 (PMC5557790; doi:10.3389/fmicb.2017.01563)
Supplement: Supplementary file 9 [file Table_3.DOC]

**Table S3. Differential abundances of bacterial genera in different age groups of cattle. Pairwise comparisons of bacterial taxa are made by metastats analysis of samples grouped according to host age: S1 (9–10 months), S2 (45–65 months), and S3 (96–120 months). Mean relative abundances of significant bacterial taxa in the three different bacterial community types are presented. Only significant taxa that contributed on average ≥0.5% to the total bacterial community in at least one community type are shown.**

| Phyla | Genera | Average relative abundance (%) | | | Significance | | |
| --- | --- | --- | --- | --- | --- | --- | --- |
|  |  | S1 (n = 6) | S2 (n = 7) | S3 (n = 7) | S1-S2 | S1-S3 | S2-S3 |
| Firmicutes | *Ruminococcus* | 5.29 ± 0.04 | 5.93 ± 0.09 | 7.45 ± 0.02 | 0.68 | 0.03 | 0.25 |
|  | *Saccharofermentans* | 2.14 ± 0.00 | 3.54 ± 0.01 | 2.71 ± 0.01 | 0.00 | 0.15 | 0.14 |
|  | *Butyrivibrio* | 2.87 ± 0.01 | 1.47 ± 0.00 | 1.43 ± 0.01 | 0.01 | 0.04 | 0.94 |
|  | *Acetivibrio* | 2.06 ± 0.02 | 0.54 ± 0.00 | 0.98 ± 0.00 | 0.01 | 0.06 | 0.05 |
|  | *Clostridium IV* | 1.17 ± 0.00 | 1.76 ± 0.01 | 2.40 ± 0.02 | 0.19 | 0.06 | 0.40 |
|  | *Cellulosilyticum* | 0.37 ± 0.00 | 0.70 ± 0.00 | 0.75 ± 0.00 | 0.11 | 0.03 | 0.82 |
|  | *Clostridium XI Va* | 0.52 ± 0.00 | 0.97 ± 0.00 | 0.82 ± 0.00 | 0.01 | 0.03 | 0.27 |
|  | *Oribacterium* | 0.98 ± 0.00 | 0.28 ± 0.00 | 0.26 ± 0.00 | 0.00 | 0.00 | 0.81 |
| Bacteroidetes | *Prevotella* | 36.89 ± 0.19 | 37.37 ± 0.41 | 27.21 ± 0.34 | 0.89 | 0.00 | 0.01 |
|  | *Tannerella* | 1.72 ± 0.01 | 2.63 ± 0.00 | 2.70 ± 0.01 | 0.04 | 0.08 | 0.89 |
|  | *Pseudozobellia* | 0.66 ± 0.00 | 0.37 ± 0.00 | 0.26 ± 0.00 | 0.17 | 0.04 | 0.39 |
| Proteobacteria | *Succinivibrio* | 4.47 ± 0.05 | 3.40 ± 0.01 | 5.54 ± 0.19 | 0.33 | 0.64 | 0.22 |
|  | *Ruminobacter* | 1.99 ± 0.03 | 2.24 ± 0.03 | 3.89 ± 0.17 | 0.81 | 0.31 | 0.39 |
| Spirochaetes | *Treponema* | 1.05 ± 0.00 | 1.39 ± 0.00 | 1.84 ± 0.01 | 0.12 | 0.05 | 0.24 |
| Fibrobacteres | *Fibrobacter* | 0.44 ± 0.00 | 1.35 ± 0.01 | 0.96 ± 0.00 | 0.01 | 0.02 | 0.29 |
| Tenericutes | *Anaeroplasma* | 0.10 ± 0.00 | 0.46 ± 0.00 | 0.70 ± 0.00 | 0.00 | 0.00 | 0.07 |
