# Supplementary material for: Role of Age-Related Shifts in Rumen Bacteria and Methanogens in Methane Production in Cattle
Source: Front Microbiol. 2017 Aug 14;8:1563. doi: 10.3389/fmicb.2017.01563 (PMC5557790; doi:10.3389/fmicb.2017.01563)
Supplement: Supplementary file 10 [file Table_4.DOC]

**Table S4.** **Abundance of archaeal taxa identified in different age groups of cattle.** S1 indicates heifers (9–10 months); S2 indicates young adults (45–65 months); S3 indicates older adults (96–120 months).

| Domain | Genera | Relative abundance (%) | | | | | |
| --- | --- | --- | --- | --- | --- | --- | --- |
|  |  | S1 (n = 6) | | S2 (n = 7) | | S3 (n = 7) | |
|  |  | Mean | SE | Mean | SE | Mean | SE |
| Euryarchaeota | *Methanobrevibacter* | 79.151 | 7.647 | 79.737 | 3.818 | 76.723 | 3.876 |
|  | *Methanomassiliicoccus* | 19.874 | 7.415 | 19.347 | 3.692 | 22.527 | 3.819 |
|  | *Methanosphaera* | 0.875 | 0.399 | 0.510 | 0.098 | 0.553 | 0.154 |
|  | *Methanobacterium* | 0.004 | 0.004 | 0.186 | 0.080 | 0.091 | 0.064 |
|  | *Methanimicrococcus* | 0.018 | 0.020 | 0.124 | 0.074 | 0.085 | 0.039 |
|  | *Methanothrix* | 0.011 | 0.006 | 0.041 | 0.022 | 0.014 | 0.012 |
|  | *Methanothermobacter* | 0.010 | 0.008 | 0.014 | 0.008 | 0.000 | 0.000 |
|  | *Methanoregula* | 0.031 | 0.029 | 0.002 | 0.001 | 0.000 | 0.000 |
|  | *Methanolinea* | 0.014 | 0.010 | 0.002 | 0.003 | 0.000 | 0.000 |
|  | *Methanocorpusculum* | 0.000 | 0.000 | 0.012 | 0.012 | 0.000 | 0.000 |
