# Supplementary material for: Role of Age-Related Shifts in Rumen Bacteria and Methanogens in Methane Production in Cattle
Source: Front Microbiol. 2017 Aug 14;8:1563. doi: 10.3389/fmicb.2017.01563 (PMC5557790; doi:10.3389/fmicb.2017.01563)
Supplement: Supplementary file 11 [file Table_5.DOC]

**Table S5. Correlations of variables with bacterial distributions at genus and OTU levels.**

| Variable | Genera | | | | Phylotype | | | |
| --- | --- | --- | --- | --- | --- | --- | --- | --- |
|  | RDA1 | RDA2 | R^2^ | P-value | RDA1 | RDA2 | R^2^ | P-value |
| Age | 0.05 | -0.99 | 0.69 | <0.01 | 0.93 | -0.37 | 0.84 | <0.01 |
| Ammonia-N | 0.13 | 0.99 | 0.11 | 0.42 | -0.90 | -0.45 | 0.24 | 0.12 |
| Acetate | -0.02 | -0.99 | 0.22 | 0.10 | 0.69 | -0.73 | 0.24 | 0.11 |
| Propionate | 0.59 | -0.81 | 0.34 | 0.03 | 0.51 | -0.86 | 0.44 | 0.02 |
| Butyrate | -0.02 | -0.99 | 0.22 | 0.10 | 0.75 | -0.66 | 0.22 | 0.13 |
| A:P ratio | -0.99 | 0.05 | 0.18 | 0.17 | -0.35 | 0.94 | 0.11 | 0.40 |
| CH_4_ (g/kg DMI) | -0.68 | 0.74 | 0.43 | <0.01 | -0.53 | 0.85 | 0.45 | <0.01 |
