# Supplementary material for: Role of Age-Related Shifts in Rumen Bacteria and Methanogens in Methane Production in Cattle
Source: Front Microbiol. 2017 Aug 14;8:1563. doi: 10.3389/fmicb.2017.01563 (PMC5557790; doi:10.3389/fmicb.2017.01563)
Supplement: Supplementary file 12 [file Table_6.DOC]

**Table S6.** OTUs related to VFAs and methane production by analysis of Sparse Partial Least Squares (sPLS) regression.

| Taxa | Abundance (%) | Regression coefficient | | | |
| --- | --- | --- | --- | --- | --- |
|  |  | Methane | Propionate | Acetate | A:P ratio |
| *Prevotella*.1 | 3.449457 | 0.027294 | -0.02346 | -0.01387 | 0.018594 |
| *Prevotella*.2 | 2.645461 | 0.011401 | -0.01958 | -0.04293 | -0.02809 |
| *Prevotella*.3 | 1.890714 | 0.010317 | 0.000713 | 0.031131 | 0.042149 |
| *Prevotella*.10 | 1.10246 | 0.029684 | -0.02031 | 0.004657 | 0.039282 |
| *Ruminococcus*.2 | 0.628102 | 0.017972 | -0.02482 | -0.04472 | -0.02212 |
| *Butyrivibrio*.1 | 0.495101 | 0.026666 | -0.01695 | 0.009122 | 0.040057 |
| *Saccharofermentans*.3 | 0.37267 | 0.037125 | -0.03319 | -0.02373 | 0.020589 |
| *Butyricicoccus*.1 | 0.355341 | 0.011416 | -0.02012 | -0.04494 | -0.03001 |
| *Prevotella*.23 | 0.313335 | 0.028413 | -0.01885 | 0.006721 | 0.039786 |
| *Ruminococcus*.5 | 0.304521 | 0.028431 | -0.01993 | 0.002644 | 0.03587 |
| *Oribacterium*.1 | 0.250068 | 0.0317 | -0.02823 | -0.01985 | 0.017978 |
| *Acetivibrio* | 0.229718 | 0.019596 | -0.02482 | -0.04025 | -0.0159 |
| *Ethanoligenens* | 0.21345 | 0.025614 | -0.02124 | -0.01008 | 0.02028 |
| *Butyrivibrio*.3 | 0.206864 | 0.034353 | -0.027 | -0.00787 | 0.032659 |
| *Moryella*.5 | 0.185121 | 0.029192 | -0.01874 | 0.009281 | 0.043171 |
| *Tannerella*.4 | 0.163676 | 0.026583 | -0.0159 | 0.012855 | 0.043564 |
| *Porphyromonas* | 0.155976 | 0.030942 | -0.02203 | 0.001588 | 0.037793 |
| *Prevotella*.41 | 0.150247 | 0.024587 | -0.01304 | 0.01824 | 0.046425 |
| *Succiniclasticum* | 0.141846 | 0.011491 | -0.01802 | -0.03676 | -0.02203 |
| *Prevotella*.44 | 0.136996 | 0.014884 | -0.02033 | -0.03619 | -0.0175 |
| *Paraprevotella*.5 | 0.131616 | 0.026872 | -0.02112 | -0.00615 | 0.025552 |
| *Anaerovorax*.1 | 0.130942 | 0.026 | -0.0225 | -0.01379 | 0.01715 |
| *Pseudobutyrivibrio*.3 | 0.124457 | 0.026216 | -0.01594 | 0.011719 | 0.042037 |
| *Butyrivibrio*.5 | 0.118717 | 0.03304 | -0.02249 | 0.005629 | 0.044153 |
| *Succinivibrio*.5 | 0.11864 | 0.008222 | -0.01679 | -0.0411 | -0.03005 |
| *Pseudoflavonifractor*.3 | 0.108801 | 0.025396 | -0.01938 | -0.00363 | 0.026253 |
| *Prevotella*.54 | 0.106048 | 0.011369 | -0.01794 | -0.03681 | -0.02222 |
| *Prevotella*.61 | 0.103839 | 0.029048 | -0.02721 | -0.02329 | 0.011549 |
| Lachnospiracea_incertae_sedis.8 | 0.101027 | 0.005698 | -0.01246 | -0.03161 | -0.02384 |
| *Ruminococcus*.1 | 2.604933 | -0.01739 | 0.025384 | 0.048475 | 0.026428 |
| *Prevotella*.16 | 0.58529 | -0.02692 | 0.025202 | 0.021534 | -0.01075 |
| *Prevotella*.21 | 0.376142 | -0.00664 | 0.014356 | 0.036213 | 0.027184 |
| *Ruminococcus*.4 | 0.347535 | -0.02585 | 0.018921 | 0.000618 | -0.0297 |
| Lachnospiracea_incertae_sedis.3 | 0.304903 | -0.02619 | 0.021523 | 0.009583 | -0.02143 |
| *Prevotella*.32 | 0.23402 | -0.02548 | 0.020964 | 0.009414 | -0.02076 |
| *Sporobacter*.2 | 0.226105 | -0.00233 | 0.012035 | 0.039291 | 0.035212 |
| *Moryella*.6 | 0.166328 | -0.01082 | 0.017836 | 0.03792 | 0.023937 |
| *Anaeroplasma* | 0.123965 | -0.02797 | 0.020628 | 0.001272 | -0.03155 |
| *Fibrobacter*.2 | 0.121686 | -0.00926 | 0.015841 | 0.034627 | 0.022579 |
| *Prevotella*.55 | 0.1125 | -0.02565 | 0.021524 | 0.011071 | -0.01936 |
| *Butyrivibrio*.4 | 0.110642 | -0.00182 | -0.00872 | -0.03813 | -0.03895 |
| Lachnospiracea_incertae_sedis.9 | 0.108695 | -0.02742 | 0.024902 | 0.019026 | -0.01376 |
| *Syntrophococcus*.1 | 0.103109 | -0.02762 | 0.025757 | 0.021701 | -0.01142 |
